# Begomovirus characterization, and development of phenotypic and DNA-based diagnostics for screening of okra genotype resistance against *Bhendi yellow vein mosaic virus*

**DOI:** 10.1007/s13205-012-0107-z

**Published:** 2012-12-20

**Authors:** V. Venkataravanappa, C. N. Lakshminarayana Reddy, M. Krishna Reddy

**Affiliations:** 1 Indian Institute of Horticultural Research, Hessaraghatta Lake PO, Bangalore, 560089 India; 2 Division of Crop Protection, Indian Institute Vegetable Research, Varanasi, 221305 Uttar Pradesh India; 3 Department of Plant Pathology, Agriculture College, GKVK Campus, University of Agricultural Sciences (B), Bangalore, 560065 India; 4 Department of Plant Pathology, College of Sericulture, University of Agricultural Sciences (B), Chintamani, 563125 Karnataka India

**Keywords:** *Bhendi yellow vein mosaic virus* (BYVMV), *Okra yellow vein* mosaic virus (OYVMV), Begomovirus, Genotypes, PCR, Susceptibility, Varieties, Resistance

## Abstract

The leaf sample from okra plants showing the yellow vein mosaic disease symptoms was collected in Karnataka state, India. The genome of the virus was amplified, cloned and sequenced. Sequence analysis revealed that the viral genome (GU112065) is 2,741 bp in length and genome is similar to that of monopartite begomoviruses originating from the Old World, with seven conserved ORFs. Further nucleotide (nts) sequence comparisons showed that the genome has the highest sequence identities of 96.1 % with *Bhendi yellow vein mosaic virus* (BYVMV) (GU112057) and 89.7 % with *okra yellow vein mosaic virus* (OYVMV) (AJ002451) infecting okra in India and Indian subcontinent. These results suggested that the isolate is a new strain of BYVMV. To identify the resistance source to BYVMV, the okra genotypes were screened under both artificial and natural conditions. None of the genotypes showed immunity to the disease. However, the genotypes Nun 1145 and Nun 1144 showed moderate resistance and genotypes M10, Nun 1142, Nun 1140 showed moderately susceptible reactions under both glass house and field conditions. Further, dot-blot hybridization using nonradioactive (digoxigenin) DNA probe showed that the virus was also detected in the symptomless plants.

## Introduction

Okra (*Abelmoschus esculentus* L.) commonly known as bhendi or lady’s finger belongs to the *Malvaceae* family and is an important vegetable crop grown across different states of the country throughout the year. Among the different species of genus, *Abelmoschus,* the most popularly grown species is *Abelmoschus esculentus* in Asia and has great commercial demand due to its nutritional value. The major production constraint for okra is yellow vein mosaic disease, causing losses with regard to the quality and as well as the yield wherever the crop is grown. The yellow vein mosaic disease of okra (YVMD) is caused by *Bhendi yellow vein mosaic virus* (BYVMV) and was first reported in 1924 from the erstwhile Bombay Presidency (Kulkarni 1924). The virus belongs to the genus *Begomovirus*, family *Geminiviridae* (Fauquet and Stanley 2005). Recently, BYVMD complex was shown to be associated with the virus with a genomic component typical of monopartite begomoviruses, homologous DNA A and a single-stranded betasatellite (Jose and Usha 2003). This species is believed to have originated from India (Usha 2008) and its only known methods of transmission are through whitefly (*Bemisia tabaci* Gennadius) and grafting. The DNA A component has seven open reading frames encoding several multifunctional proteins involved in rolling circle replication, gene transcription, cell-to-cell and long-distance movement, suppression of host gene silencing, and encapsidation of the viral genome (Lazarowitz 1992). Betasatellites are approximately half the size of their helper begomoviruses required to induce typical disease symptoms in their original hosts (Briddon et al. 2001, 2003; Jose and Usha 2003). These satellites depend on their helper virus for replication, movement, encapsidation and vector transmission. The YVMD is characterized by a homogenous interwoven network of yellow vein enclosing islands of green tissue within its leaf. In extreme cases, infected leaves become completely yellowish or creamy. If plants are infected within 20 days after germination, their growth is retarded with few leaves and malformed fruits resulting in loss ranging from 94 to 100 % (Pun and Doraiswamy 1999). The extent of damage declines with delay in infection of the plants and was reported with a loss of 49–84 %, when infection occurred after 50–65 days of germination (Sastry and Singh 1974).

Further, the decline in the production of okra in India was attributed to several factors, such as loss of resistance to yellow vein mosaic in ruling varieties (Borah et al. 1992), emergence of new biotypes of whitefly vectors and development of moderate to strong resistance to commonly used insecticides by vectors (Rashida et al. 2005). At this stage, in order to implement sustainable pest management practices for the okra cropping system, there is a need to come out with tools, which will aid in quick identification of the virus/strains of begomovirus associated with yellow vein mosaic disease and to screen okra germplasm for YVMD resistance. With this backdrop, the current study was aimed at virus characterization and development of phenotypic and DNA-based diagnostics for screening germplasm to address this issue.

## Materials and methods

### Virus isolate and maintenance of *Bemisia tabaci*

A leaf sample from okra plants showing prominent yellow vein mosaic symptoms and two samples from non-symptomatic plants were collected from Chintamani, Karnataka state, India. The YVMD from the sample was whitefly transmitted to susceptible okra genotype (*cv*. 1685) and designated as virus isolate OYCHINT. Whitefly collection, maintenance and transmission were carried out as described by Venkataravanappa et al. (2012). After transmission, the inoculated plants were sprayed with an insecticide and maintained under insect-proof glasshouse for symptom expression. The plant tissues showing the symptoms were utilized for further analysis.

### DNA isolation, PCR amplification, cloning and sequencing

Total nucleic acids were extracted from both non-symptomatic and symptomatic leaf tissues by the Cetyl trimethyl ammonium bromide method (Doyle and Doyle, 1990). The different components of virus genome were amplified by PCR as per the protocol and primers described by Venkataravanappa et al. (2012). The amplicons were cloned into the pTZ57R/T vector (Fermentas, Germany) according to the manufacturer’s instructions. The complete nucleotide sequence of the clones were determined by automated DNA sequencer ABI PRISM 3730 (Applied Biosystems) at Anshul Biotechnologies DNA Sequencing facility, Hyderabad, Andhra Pradesh, India. Three clones for each fragment were subjected to sequencing.

### Comparison of DNA sequence

The similarity of genomic sequences was initially analyzed by using the BLAST program available at the National Center for Biotechnology Information (http://www.ncbi.nlm.nih.gov/BLAST/). The sequences (Table 1) showing highest scores with the present isolate were obtained from the database and multiple aligned using CLUSTAL-X program (Thompson et al. 1994). The sequence identity matrixes were generated using Bioedit Sequence Alignment Editor (version 5.0.9) (Hall, 1999) and phylogenetic tree was generated by MEGA 5.0 software (Tamura et al. 2011) using the neighbor joining method with 1,000 bootstrapped replications.

**Table 1:** GenBank accession numbers of selected begomovirus sequences from Asia used in this study for analysis

Begomoviruses	Accession number	Abbreviation
Cotton leaf curl Multan virus—Hisar [Pakistan:Multan 311:Okra:96]	AJ002459	CLCuMVHis[PK:M311:Ok:96]
Mesta yellow vein mosaic Bahraich virus—[India:Bhanga:2008]	FJ159268	MeYVMV-[IN:Bongaon:08]
Cotton leaf curl Shadadpur virus—[Pk:Sindh,Tjam:09]	FN552002	CLCuSV[PK:Tjam:05]
Bhendi yellow vein mosaic virus—[India:Himachal:2010]	FR694925	BYVMV-[IN:Him:2010]
Bhendi yellow vein mosaic virus—[India:Kaivara:OYKaivara:06]	GU112057	BYVMV[IN:Kai:OYKaivara:2006]
Bhendi yellow vein mosaic virus—[India:Raichur:OY49:05]	GU112066	BYVMV[IN:Rair:OY49:05]
Bhendi yellow vein mosaic virus—[India:Kerala:OYG6AG:05]	GU112060	BYVMV[IN:Ker:OYG6AG:05]
Bhendi yellow vein mosaic virus—[India:Chelur:OYCN6:06]	GU112059	BYVMV[IN:Chelr:OYCN6:06]
Bhendi yellow vein mosaic virus—[India:Sonipat:OY93:05]	GU112061	BYVMV[IN:Sonit:OY93:05]
Bhendi yellow vein mosaic virus—[India:Varanasi:OY35:05]	GU112056	BYVMV[-[IN:Var:OY35:05]
Bhendi yellow vein mosaic virus—[India:Phalaghat:OY014:05]	GU112055	BYVMV[IN:Phal:OY014:05]
Bhendi yellow vein mosaic virus—[India:Kaivara:OYKaivara1:06]	GU112058	BYVMV[IN:Kai:OYKaivara1:06]
Bhendi yellow vein mosaic virus—[India:New Delhi:OY134:05]	GU112063	BYVMV[IN:ND:OY134:05]
Bhendi yellow vein mosaic virus—[India:Phalaghat:OY07:05]	GU112062	BYVMV[IN Phal:OY07:05]
Bhendi yellow vein mosaic virus—[India:New Delhi:OY133:06]	GU112078	BYVMV[IN:ND:OY133:06]
Bhendi yellow vein mosaic virus—[India:Pandarahalli:OY167:06]	GU112079	BYVMV[IN:Pand:OY167:06]
Bhendi yellow vein mosaic virus—[India:Raichur:OY59:05]	GU112070	BYVMV[IN:Rai:OY59:05]
Bhendi yellow vein mosaic virus—[India:Pandarahalli:OY174:06]	GU112073	BYVMV[IN:Pand:OY174:06]
Bhendi yellow vein mosaic virus—[India:Tirupathi:OY99:05]	GU112071	BYVMV[IN:Tiri:OY99:05]
Bhendi yellow vein mosaic virus— [India:Phalaghat:OY138A:06]	GU112072	BYVMV[IN:Phal:OY138A:06]
Bhendi yellow vein mosaic virus—[India:Raichur:OY56:05]	GU112074	BYVMV[IN:Rai:OY56:05]
Bhendi yellow vein mosaic virus—[India:Sonipat:OY83:05]	GU112075	BYVMV[IN:Soni:OY83:05]
Bhendi yellow vein mosaic virus—[India:Raichur:OY54A:05]	GU112067	BYVMV[IN:Rai:OY54A:05]
Bhendi yellow vein mosaic virus— BYVMV[India:Raichur:OY54B:05]	GU112068	BYVMV[IN:Rair:OY54B:05]
Bhendi yellow vein mosaic virus—[India:Bhemarayanagudi:OY45:05]	GU112069	BYVMV[IN:Bhe:OY45:05]
Bhendi yellow vein mosaic virus –[India:Karnal:OY80B:06]	GU112077	BYVMV[IN:Kar:OY80B:06]
Bhendi yellow vein mosaic virus—[India:Coimbator:OYCO4:05]	GU112080	BYVMV[IN:Coi:OYCO4:05]
Bhendi yellow vein mosaic virus—[India:Karnal:OY79A:05]	GU112076	BYVMV[IN:Kar:OY79A:05]
Bhendi yellow vein mosaic virus—India [India:Madurai]	AF241479	BYVMV-IN[IN:Mad]
Bhendi yellow vein mosaic virus—[India:Bangalore:OY34:05]	GU112064	BYVMV[IN:Ban:OY34:05]
Bhendi yellow vein mosaic virus—[India:Chintamani:OYCHINT:06]	GU112065	BYVMV[I IN:Chint:OYCHINT:06]
Bhendi yellow vein mosaic virus—[India:Guntur OY112:05]	GU112005	BYVMV[IN:Gun:OY112:05]
Bhendi yellow vein mosaic virus—[India:Sonipat EL13:2006]	GU112007	BYVMV-[India:Soni:EL13:2006]
Bhendi yellow vein mosaic virus—[India:Sonipat OY92B:2005]	GU112006	BYVMV-[India:Soni:OY92B:2005]
Bhendi yellow vein mosaic virus—[India:Haryana:2009]	FN645917	BYVMV-[India:Har:2009]
Bhendi yellow vein mosaic virus.NOL751—India [India:Maharashtra]	EU589392	BYVMV NOL751-IN[IN.Maha:08]
Okra yellow vein mosaic virus—[Pakistan:Faisalabad 201:95]	AJ002451	OYVMV-[PK:Fai201:95]
Bhendi yellow vein mosaic virus—Pakistan [Pakistan:Multan301:96]	AJ002453	BYVMVPK[PK:M301:96]
Tomato leaf curl New Delhi virus—India [India:New Delhi:AVT1]	AY428769	ToLCNDVIN[IN:ND:AVT1]
Tomato leaf curl New Delhi virus—[India:Aurangabad:okra:06]	GU112088	ToLCNDV.[IN:Aur:OY164A:06]
Tomato leaf curl New Delhi virus—[India:Karnal:okra:04]	GU112082	ToLCNDV.[IN:Kar:OY81A:04]
Tomato leaf curl New Delhi virus—[India:Guntur:okra:06]	GU112086	ToLCNDV.[IN:Gun:OY136B:06]

### Plant material

The okra germplasm used for screening in the present study was the popularly grown tolerant variety/hybrid obtained from different sources. They are Arka Anamika, Pusa Sawani, cv1685, VRO-6, Punjab7, Hyb.218, HRB-107-4, AC1605(H5), NS 98, Nun 1144, Nun 1145, Nun 1142, Nun 1143, Nun 1140, M10, Kanchan and Indol 03-1. Apart from these some of the advanced breeding lines of okra viz; A.AXDJM-32, A.AX IIHR-1, DJM-32 X *A. tetraphyllus*, DJMA-3, IIHR-1XA.A, IIHR-233, IIHR-222, IC-141055, PKt3S3, PKt5S7, PKt6S6, PKt12S6 were collected from Division of vegetable breeding, Indian Institute of Horticulture Research, Bangalore, Karnataka.

### Glasshouse screening of okra genotypes by whitefly inoculation

Inoculation of begomovirus by *B. tabaci* was conducted using cylindrical cages with mesh tops which were inverted over individual leaves. The insects were given access to YVMD-infected okra plants maintained in glass house in separate whitefly-proof cages. After acquisition access period of 24 h, the whiteflies were collected individually using an aspirator and transferred to separately caged test plants. Ten viruliferous adult whiteflies per each 1-week-old test plant were released and 24 h inoculation access period was given. After that the whiteflies were removed and plants were sprayed with 0.05 % imidacloprid insecticide and maintained in insect-proof screen house for symptom development. In each genotype, five plants were inoculated with nonviruliferous whiteflies, which were given acquisition access to healthy plants, which served as a control.

### Natural screening of okra genotypes under field condition

A total of 20 genotypes of okra was screened for YVMD along with the susceptible check okra cultivar, 1685. For, every four rows of test genotype, one row of susceptible check were planted. Disease incidence was recorded and calculated using the formula below:*a* is the number of diseased plants and *b* is the number of healthy plants.

### Genotype classification

The okra varieties/hybrid/line were classified based on disease response to YVMD under both artificial and natural conditions using criteria previously described by Borah et al. (1992).

Grouping of plant response to infection of begomovirus.

**Table:** 

S. no.	Disease incidence (%)	Plant response
1	0.0	Immune
2	*X* < 10	Highly resistant (HR)
3	10 < *X* > 20	Resistant (R)
4	20 < *X* > 30	Moderately resistant (MR)
5	30 < *X* > 50	Moderately susceptible (MS)
6	50 < *X* > 70	Susceptible (S)
7	*X* > 70	Highly susceptible (HS)

### DNA probe labeling, dot-blot hybridization and colorimetric detection

Coat Protein gene on homologous DNA A component of BYVMV (Isolate OYCHINT, Acc. No. GU112065) was used to design the probe. PCR amplification of coat protein gene was carried out with specific primer CPF and CPR and the amplified fragment was purified using the QIAquick gel extraction kit (QIAGEN Inc. Valencia, CA). The amplicon was then subjected for labeling using Random Primed Labeling with DIG-High Prime kit II (Roche diagnosis, Germany). Total nucleic acids were extracted from both symptomatic and non-symptomatic plants of different okra genotypes as described above. Total DNA of 20 μl from each sample was heated for 5 min at 100 °C on water bath and incubated at 4 °C before loading onto the membrane. After cooling, the DNA was loaded onto nitrocellulose membrane (Hybond-N, Amersham) using membrane loading commercial device (Dot Blot 96 System, Biometra, Germany). Then the membrane was air dried and DNA was cross-linked to the membrane by exposing to ultraviolet light (in a crosslinker device Amersham Pharmacia Biotech, USA). Different dilutions (10^−1^, 10^−2^, 10^−3^ and 10^−4^) of nucleic acid were used to check sensitivity of hybridization technique to detect the begomovirus in okra samples.

The pre-hybridization, hybridization and detection procedures were carried out according to the protocol given in DIG-High Prime DNA labeling and detection starter kit II (Roche diagnostics). Colorimetry-based detection was done with the use of nitroblue tetrazolium (NBT) and X-phosphate. Development of purple color at the location of sample on nitrocellulose membrane was indicative of positive reaction.

## Results and discussion

### PCR amplification, genome organization and sequence analysis

The complete genome component of virus isolate OYCHINT was amplified from the okra samples infected with YVMD collected from the field as well as glasshouse using three sets of primers. These primers were designed to amplify full genome in three fragments with approximately more than 200 bp overlapping to rule out mixed infections. Amplification with nucleic acid extracts from symptomless plants yielded no product. Positive amplification to betasatellite and failed to confirm the association of DNA B provided the evidence to conclude that, the current isolate under study is monopartite begomovirus.

The genome sequence of homologous DNA A component of the virus isolate was determined in both orientations and it was found to be 2,741 nucleotides in length and the sequence is available in the database under the accession number GU112065. The sequence had features, typical of Old World monopartite begomoviruses, with two open reading frames (ORFs) [AV1 (CP), AV2] in virion-sense strand and five ORFs [AC1 (Rep), AC2, AC3, AC4, AC5] in complementary-sense strand separated by an intergenic region (IR). In the IR region, the sequence identity of virus isolate was more than 90 % with IRs of BYVMV, for which a full-length sequence is available in the databases. The length of intergenic region (IR) is 298 nucleotides and encompasses an absolutely conserved hairpin structure containing nonanucleotide sequence (TAATATTAC) that marks the origin of virion-strand DNA replication and with repeated sequences known as “iterons” (GGAGTC) adjacent to the TATA box, which is the recognition sequence for binding of the rep to the promoter (Arguello-Astorga and Ruiz-Medrano 2001; Hanley-Bowdoin et al. 1999).

**Fig. 1:**
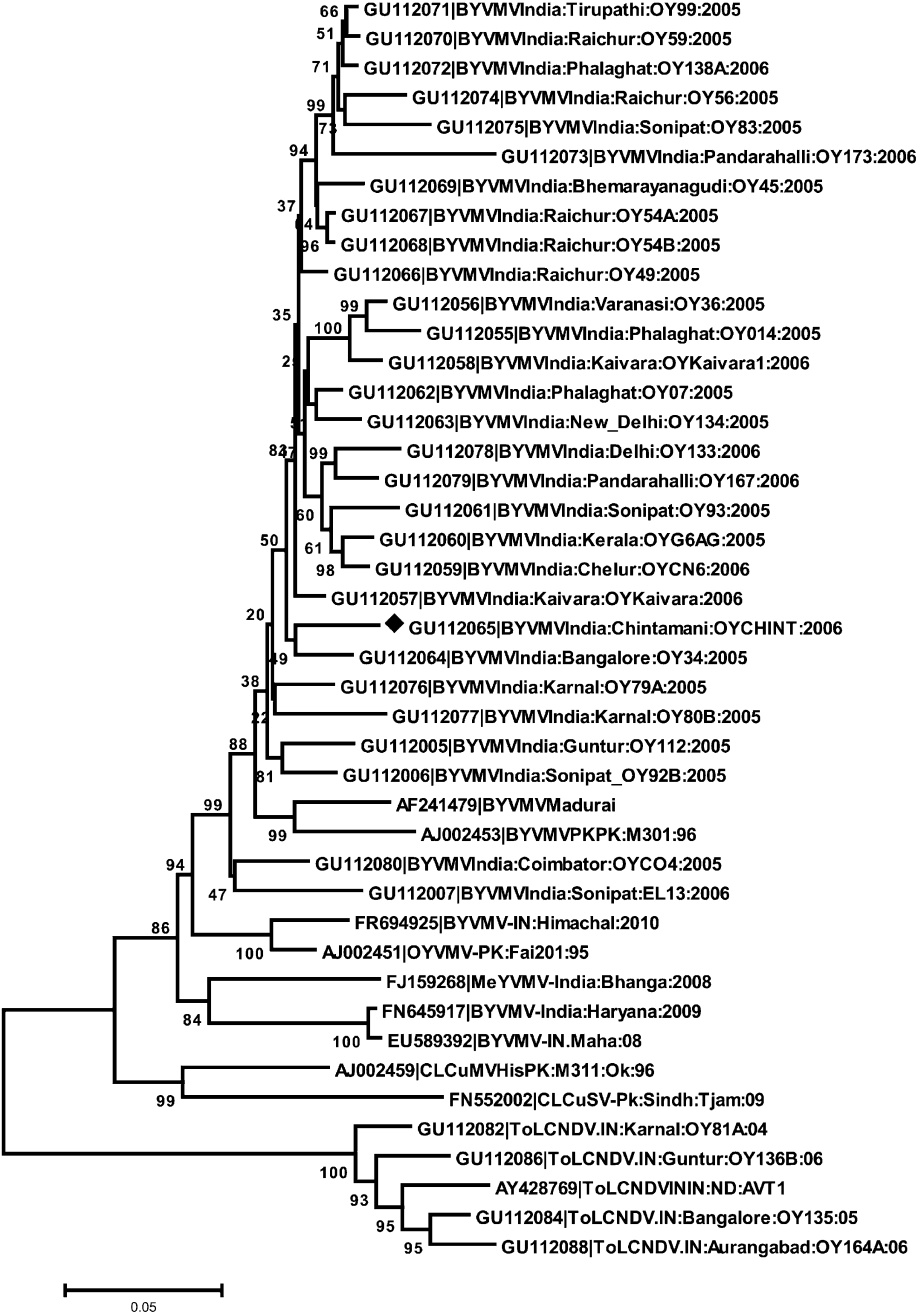
Phylogenetic trees constructed from aligned complete genome sequence (homologous DNA A component) of OYCHINT virus isolate with other begomoviruses by MEGA 5 using Neighbor-joining algorithm.*Horizontal* distances are proportional to sequence distances,*vertical* distances are arbitrary. The trees are unrooted. A bootstrap analysis with 1,000 replicates was performed and the bootstrap percent values more than 50 are numbered along branches

The comparison of genome sequence with the selected begomovirus sequences revealed that it shared highest sequence identity of 96.1 % with BYVMV (GU112057) and 89.7 % with OYVMV-(AJ002451) infecting okra in India and Indian subcontinent. This result was well supported by phylogenetic analysis with OYCHINT isolate closely clustering with BYVMV group (Fig. 1). Based on the current taxonomic criteria for begomovirus, the threshold cutoff of nucleotide identity for species demarcation is 89 % (Fauquet et al. 2008) and the virus isolates displaying more than this should be considered as strains rather than different virus species (Padidam et al. 1995). The present results indicate that OYCHINT is a new strain of *Bhendi yellow vein mosaic virus* from India infecting okra.

### Evaluation of okra genotypes

#### Screening of germplasms under artificial condition

**Fig. 2:**
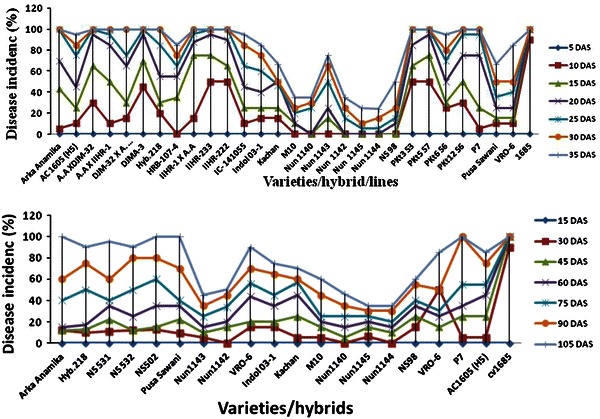
**a** YVMD disease incidence in okra genotype under artificial condition at different time intervals. **b** YVMD disease incidence in okra genotype under natural condition at different time intervals

Totally, twenty-nine genotypes were screened under artificial condition by whitefly inoculation. The visual symptoms developed on different inoculated okra genotypes varied from yellow vein mosaic, vein thickening, petiole bending, venial chlorosis, intense yellowing, later turned brown, premature death and stunted growth of plant (Table 2). The disease incidence among different inoculated okra genotypes ranged from 5 to 100 %. Based on their susceptible reaction, the genotypes were classified as being resistant to highly susceptible by using criteria as previously described by Borah et al. (1992). None of the genotypes were immune to the virus. However, genotypes Nun 1145 and Nun 1144 showed moderate resistance reaction and symptoms appeared after 25–30 days of inoculation. Whereas, the genotypes Nun 1142, Nun 1140, and M10 showed moderately susceptible reaction and symptoms were produced after 15–25 days after the inoculation (Fig. 2a; Table 2). The genotypes Arka Anamika and Pusa sawani earlier showed to be resistant to BYVMV (Borah et al. 1992) were highly susceptible with much faster development of disease symptoms than other tested genotypes. The variation in symptoms in genotypes may be due to unique interaction between the particular virus strain and plant genotype or vector and genotype or altered feeding conditions of the vector (Polston and Anderson 1997; Delatte et al. 2006; Azizi et al. 2008). The begomoviruses transmitted by whitefly are directly deposited into phloem during salivation. Therefore, altered feeding behavior could result in a significant diminishing in the incidence of several begomoviruses that are usually interpreted as being resistant to insect vector (Parejarearn et al. 1984; Dintinger et al. 2005; Azizi et al. 2008).

**Table 2:** Responses of okra genotypes/lines to the infection of yellow vein mosaic disease of okra under artificial conditions

Varieties/hybrids/lines	Ti/T^a^	Incubation period	Disease incidence (%)	Type of symptoms	Plant response
A.A X IIHR-1	20/20	8–10	100.0	Intense yellowing, petiole bending, vein netting	HS
A.A XDJM-32	20/20	8–10	100.0	Yellow vein, stunted growth and minute enation	HS
AC 1605 (H5)	20/19	8–10	95.00	Vein clearing, veinal chlorosis and petiole bending	HS
Arka Anamika	20/20	8–10	100.0	Vein clearing, veinal chlorosis, complete yellowing	HS
DJM-32 X *A. tetraphyllus*	20/20	8–10	100.0	Intense yellowing, vein netting	HS
DJMA-3	20/20	8–10	100.0	Intense yellowing, petiole bending, vein netting	HS
HRB-107-4	17/20	12–15	85.00	Yellow vein mosaic, vein thickening	HS
Hyb.218	20/20	8–10	100.0	Yellow vein mosaic	HS
IC-141055	19/20	8–10	95.00	Yellow vein mosaic	HS
IIHR-1 X A.A	20/20	8–10	100.0	Yellow vein mosaic, vein thickening, petiole bending	HS
IIHR-222	20/20	8–10	100.0	Yellow vein mosaic, vein thickening, petiole bending	HS
IIHR-233	20/20	8–10	100.0	Yellow vein mosaic, vein thickening	HS
Indol 03-1	17/20	8–10	85.00	Yellow vein mosaic, vein thickening, petiole bending	HS
Kachan	7/20	8–10	66.60	Yellow vein mosaic, vein thickening	HS
M10	7/20	15–20	35.00	Yellow vein mosaic	MS
NS 98	10/20	19–20	50.00	Vein clearing, veinal chlorosis, stunted growth	HS
Nun 1142	7/20	20–25	35.0	Yellow vein mosaic	MS
Nun 1145	5/20	25–30	24.0	Yellow vein mosaic	MR
Nun 1140	7/20	23–25	35.00	Vein clearing, veinal chlorosis, stunted growth	MS
Nun 1143	15/20	12–15	75.00	Yellow vein mosaic	HS
Nun 1144	5/20	25–30	25.0	Yellow vein mosaic	MR
Okra cv.1685	20/20	8–10	100.0	Yellow vein mosaic, vein twisting, downward curling	HS
P7	20/20	8–10	100.0	Yellow vein mosaic, complete yellowing	HS
PKt12 S6	20/20	8–10	100.0	Yellow vein mosaic and vein netting	HS
PKt3 S3	20/20	8–10	100.0	Yellow vein mosaic, complete yellowing, stunted growth	HS
PKt5 S7	20/20	8–10	100.0	Yellow vein mosaic, complete yellowing, enation	HS
PKt6 S6	19/20	8–10	95.0	Yellow vein mosaic, complete yellowing, stunted growth	HS
Pusa Sawani	20/20	8–10	100.0	Yellow vein mosaic, complete yellowing	HS
VRO-6	17/20	8–10	85.00	Yellow vein netting, stunted plant growth	HS

Inoculation was carried out by using whitefly with a 24-h acquisition feeding period, a 24-h inoculation feeding period. Ten flies per plant were used

^a^Number of plants showing symptom (Ti)/total number of plants tested (T)

#### Screening of germplasm under natural condition

Twenty okra genotypes were evaluated under field conditions with some advanced okra breeding lines. Three types of visual symptoms were observed on different okra genotypes. First type, the leaves of the young plants infected very early in the season became complete yellow and the leaves later turned brown and dried up. In the second type, plant infection started after flowering, upper leaves and flowering parts showed vein clearing symptoms. Infected plants produced some fruits but they became yellow and hard at picking stage. Third type, plants continued to grow in a healthy state and fruiting was normal till late in the season but, at the end, few small young shoots appeared at the basal portion of the stem, which showed only vein clearing. However, in such plants yield was as good as symptomless plants. This variation in symptoms under natural condition may be due to many factors like virus strain, time of infection, plant genotype, variation in biotypes of vector and their transmission and environmental factors (Polston and Anderson, 1997). The response of the varieties to virus infection under natural condition varied from incidence of 5 to 100 %. Further, their reaction of genotypes was classified similar to above. The genotypes Nun 1145 and Nun 1144 showed moderate resistance and genotypes Nun 1140, Nun 1142, Nun 1143 and M10 showed moderately susceptible reaction under natural condition (Fig. 2b; Table 3).

**Table 3:** Responses of okra genotypes to the infection of yellow vein mosaic disease of okra under natural conditions

Varieties/hybrids	Disease incidence days after sowing		Type of symptoms	Plant response	Hybridization reaction
	15	105			
AC1605 (H5)	0.00	85.00	Vein clearing, veinal chlorosis, complete yellowing	HS	NT
Arka Anamika	0.00	100.00	Vein clearing, veinal chlorosis, enation on leaves	HS	NT
Hyb.218	0.00	90.00	Yellow vein mosaic and malformed fruits	HS	NT
Indol 03-1	0.00	75.00	Complete yellowing, stunted growth and minute enation	HS	NT
Kachan	0.00	70.00	Vein clearing, veinal chlorosis, petiole bending	HS	NT
M10	0.00	60.00	Yellow vein mosaic	MS	++
NS 531	0.00	95.80	Vein clearing, veinal chlorosis, and petiole bending	HS	NT
NS 532	0.00	89.40	Vein clearing, Veinal chlorosis	HS	NT
NS 502	0.00	100.00	Yellow vein mosaic	HS	NT
NS 98	0.00	60.00	Vein clearing, veinal chlorosis, stunting of plants	HS	NT
Nun 1140	0.00	36.00	Yellow vein mosaic	MS	++
Nun 1142	0.00	50.00	Vein netting, yellow vein mosaic	MS	++
Nun 1143	0.00	46.00	Vein clearing, veinal chlorosis and enation	MS	++
Nun 1144	0.00	35.00	Yellow vein mosaic, vein chlorosis	MR	+
Nun 1145	0.00	35.00	Vein clearing, veinal chlorosis, malformed fruits	MR	+
Okra cv1685	0.00	100.0	Vein clearing, veinal chlorosis, petiole bending	HS	+++
P7	0.00	100.0	Yellow vein, malformed fruits and enation	HS	NT
Pusa Sawani	0.00	100.00	Intense yellowing, veinal chlorosis, malformed fruits	HS	NT
VRO-6	0.00	90.00	Vein clearing, veinal chlorosis, complete yellowing	HS	NT
VRO-6	0.00	85.00	Vein clearing, veinal chlorosis, malformed fruits	HS	NT

*NT* not tested because all the plants were showing the symptoms

Reaction of hybridisation: +++strong, ++ moderate, + weak

^a^Number of plants showing symptom (Ti)/total number of plants tested (T)

Based on the number of plants showing symptoms, both under artificial and natural conditions, cv. Nun 1144 and Nun 1145 were found to be moderately resistant, M10, Nun 1140, Nun 1142 and Nun 1143 were found to be moderately susceptible to the virus, whereas other genotypes were found to be susceptible to highly susceptible. Although completely resistant genotypes were not observed in this study, few genotypes which demonstrated tolerant-like responses to the virus infection can be utilized in breeding programmes. Certain genotypes such as Nun 1144 Nun 1145, M10, Nun 1140 and Nun 1142 have longer incubation period and fewer (<50 %) infected plants when inoculated with the virus. Similar results were observed when screening of different okra genotypes resistant to *Bhendi yellow vein mosaic virus* in the earlier studies (Dhankhar et al. 1996; Srivastava et al. 1995; Sannigrahi and Choudhury 1998; Batra and Singh 2000).

### Dot-blot hybridization for detection of yellow vein mosaic virus

**Fig. 3:**
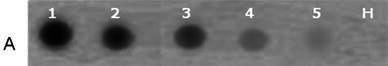
Detection of begomovirus from okra plants using digoxigenin-labeled DNA probe with dot-blot hybridization method. *A 1* DNA-clone of BYVMV, *2A* infected sample, *3*–*5* plant extract from plants showing symptoms after dilution factor of 10^−1^, 10^−2^, 10^−3^ respectively, *H* healthy sample

**Fig. 4:**
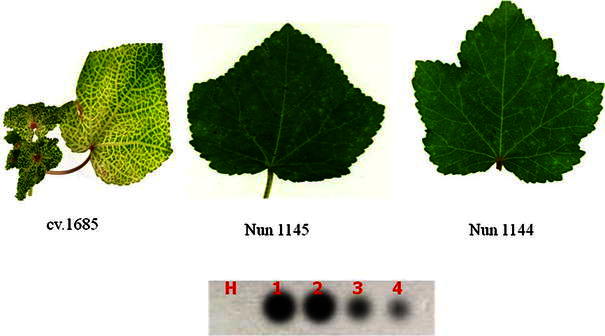
Detection of begomovirus from okra plants using digoxigenin-labeled DNA probe with dot-blot hybridization method. *H* healthy control, *1* +VE clone of BYVMV, *2* positive control (cv.1685), *3* moderately resistant (Nun 1145), *4* moderately resistant (Nun 1144)

The non-radioactive digoxigenin-labeled DNA probe was used in dot-blot hybridization to detect the virus in the total DNA isolated from the symptomatic plant as well as non-symptomatic plant of different okra genotypes. The probe could be detected up to a concentration of 10^−2^ dilution in the plant showing the yellow vein mosaic disease (Fig. 3). The symptomless okra plants in all genotypes screened were also showed positive reaction. However, in certain genotypes the intensity of the reaction was less when compared to the plants expressing the symptoms. Based on the intensity of the reaction, the detection level could be differentiated into weak to strong reaction which is in turn indicative of virus titer in the host plants (Table 4; Fig. 4). Virus titer in plant tissue is an indicator of resistance or susceptibility of plants to the virus. Low levels of virus titer and decreasing virus accumulation rate in plant tissue indicate the presence of a resistance mechanism in the plant (Pico et al. 2001; Lapidot et al. 1997; Romero-Durban et al. 1993; Sharma et al. 2004). In the present study despite the high levels of similarities in symptom development in all the genotypes, there were considerable differences in BYVMV concentration in two genotypes (Nun 1144 and Nun 1145) both under artificial and natural conditions. Therefore, we tried to estimate the virus titer in both symptomatic and symptomless okra plants using digoxigenin-labeled DNA probe, the probe could detect begomovirus in both symptomatic and non-symptomatic plants (Table 4). Although radioactive methods have been widely used for several purposes including plant viral detection (Rodriguez et al. 2003), the introduction of non-radioactive probes has been necessary due to the environmental and technological disadvantages of the radioactive probes. Several authors have reported the use of the non-radioactive probe using markers such as digoxigenin, biotin and photobiotin, which are able to detect viral concentrations as low as compared to the radioactive probes (Singh et al. 1994; Li et al. 1995; Romero-Durban et al. 1993; Nakahara et al. 1998). Based on the outcome of the present study, it can be concluded that potential application of nonradioactive DNA probe for determining actual response of plant genotypes can be useful for routine large-scale diagnosis of geminiviruses affecting economically important crops in India.

**Table 4:** Detection of begomovirus infection in okra varieties/hybrids/lines using dot-blot hybridization

Varieties/hybrids/lines	Plant showing symptom		Plant with no symptom	
	Number	Hybridization reaction	Number	Hybridization reaction
A.A X IIHR-1	20	TD	0	NT
A.A XDJM-32	20	TD	0	NT
AC 1605 (H5)	19	+++	1	+++
Arka Anamika	20	TD	0	NT
cv. 1685	20	TD	0	NT
DJM-32 X *A. tetraphyllus*	20	TD	0	NT
DJMA-3	20	TD	0	NT
HRB-107-4	17	+++	3	+++
Hyb.218	20	TD	0	NT
IC-141055	19	+++	1	+++
IIHR-1 X A.A	20	TD	0	NT
IIHR-222	20	TD	0	NT
IIHR-233	20	TD	0	NT
Indol 03-1	17	+++	3	+++
Kachan	7	++	13	++
M10	7	++	13	++
NS 98	10	++	10	++
Nun 1142	7	++	13	++
Nun 1145	5	++	15	+
Nun 1140	7	++	13	++
Nun 1143	15	++	5	++
Nun 1144	5	++	15	+
P7	20	TD	0	NT
PKt12 S6	20	TD	0	NT
PKt3 S3	20	TD	0	NT
PKt5 S7	20	TD	0	NT
PKt6 S6	19	+++	1	+++
Pusa Sawani	20	TD	0	NT
VRO-6	17	+++	3	+++

*NT* not tested because all the plants were showing the symptoms

Reaction of hybridisation: +++ strong, ++ moderate, + weak

*TD* Total disease
